# Human Cytomegalovirus Upregulates Expression of HCLS1 Resulting in Increased Cell Motility and Transendothelial Migration during Latency

**DOI:** 10.1016/j.isci.2019.09.016

**Published:** 2019-09-14

**Authors:** Yusuf Aslam, James Williamson, Veronika Romashova, Elizabeth Elder, Benjamin Krishna, Mark Wills, Paul Lehner, John Sinclair, Emma Poole

**Affiliations:** 1 Cambridge University, Department of Medicine, Level 5, Addenbrooke's Hospital, Hills Road, Cambridge CB2 0QQ, UK; 2 Lerner Research Institute, Cleveland Clinic, Cleveland, OH 44106, USA

**Keywords:** Cellular Physiology, Immunology, Virology

## Abstract

Human cytomegalovirus establishes a lifelong, latent infection in the human host and can cause significant morbidity and mortality, particularly, in immunocompromised individuals. One established site of HCMV latency and reactivation is in cells of the myeloid lineage. In undifferentiated myeloid cells, such as CD14+ monocytes, virus is maintained latently. We have recently reported an analysis of the total proteome of latently infected CD14+ monocytes, which identified an increase in hematopoietic lineage cell-specific protein (HCLS1). Here we show that this latency-associated upregulation of HCLS1 occurs in a US28-dependent manner and stabilizes actin structure in latently infected cells. This results in their increased motility and ability to transit endothelial cell layers. Thus, latency-associated increases in monocyte motility could aid dissemination of the latently infected reservoir, and targeting this increased motility could have an impact on the ability of latently infected monocytes to distribute to tissue sites of reactivation.

## Highlights

• HCLS1 is upregulated in cells latently infected with HCMV
• HCLS1 modulates monocyte motility
• Latent infection with HCMV increases monocyte motility

## Graphical Abstract

**Figure:**
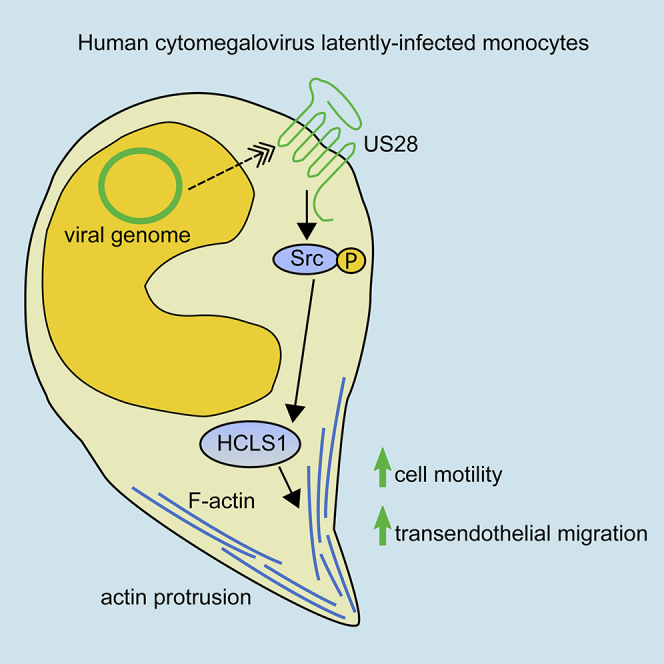

## Introduction

Following primary infection with human cytomegalovirus (HCMV), the virus is never cleared but is carried for life. This lifelong viral persistence is underpinned by true latent carriage in certain cell types *in vivo,* which sporadically reactivate subclinically (Poole and Sinclair, 2015, Sinclair and Poole, 2014). In normal healthy carriers, HCMV primary infection or reactivation is rarely symptomatic, but it does cause significant morbidity and mortality in the immunocompromised, immunosuppressed, or the immunonaive. One established site of HCMV latency *in vivo* is known to be CD34+ progenitor cells of the myeloid lineage (Poole and Sinclair, 2015, Sinclair and Poole, 2014). For instance, CD34+ progenitor cells from the bone marrow or from granulocyte colony-stimulating factor-mobilized donors have been shown to carry viral genome in the absence of detectable virus production (Poole and Sinclair, 2015, Reeves et al., 2005a, Reeves et al., 2005b, Sinclair and Poole, 2014), an accepted hallmark of latent infection. It is now also clear that CD14+ monocytes, which are derived from CD34+ progenitors, also carry latent viral genomes. However, as these myeloid cells differentiate to tissue macrophages and dendritic cells (DCs), virus reactivates resulting in lytic infection and the *de novo* production of infectious virions. The effect of latent infection on myeloid cells has now become a topic of considerable interest, and, far from the view that latency is a passive carriage of quiescent viral genomes, more recent studies suggest that latent infection imparts important changes on the cell, which support maintenance of latency and enable efficient virus reactivation (Krishna et al., 2016, Lau et al., 2016, Mason et al., 2012, Poole et al., 2014, Poole et al., 2015, Poole et al., 2011, Poole and Sinclair, 2015, Rossetto et al., 2013, Slobedman and Cheung, 2008). For instance, studies using experimental models of latency have shown that latent infection of myeloid cells with HCMV profoundly modulates the cell secretome, apoptome, and microRNAome (Mason et al., 2012, Poole et al., 2011, Poole et al., 2014, Poole et al., 2015, Poole and Sinclair, 2015, Rossetto et al., 2013, Slobedman and Cheung, 2008). Recently, we reported an analysis of total latency-associated changes in the cell proteome of latently infected CD14+ monocytes using Tandem Mass Tag technology and identified robust changes in cellular proteins resulting from latent infection (Elder et al., 2019). Besides the secreted cellular proteins S100A8 and A9, which we have already reported on (Elder et al., 2019), one of the other most highly upregulated proteins was hematopoietic cell lineage-specific protein 1 (HCLS1). HCLS1 has been implicated in a number of cellular processes, but its role in cell motility, centered on actin rearrangement, is well established. For instance, HCLS1 is a cortactin homolog and can increase the stability of actin filaments (Cavnar et al., 2012, Dehring et al., 2011, Gomez et al., 2006, Hao et al., 2005, Mukherjee et al., 2015, Uruno et al., 2003). Interestingly, HCMV is known to regulate actin at a number of points in lytic infection, and this helps to mediate viral egress (Wilkie et al., 2016), restructure lipid rafts (Low et al., 2016), impair immune recognition (Fielding et al., 2014, Gabaev et al., 2014), and promote cell migration (Dehring et al., 2011, Reinhardt et al., 2014, Streblow et al., 1999, Tseliou et al., 2016). However, little is known about the effect of latent infection on actin, although it is known that virus binding to monocytes can cause immediate effects on paxillin protein, which regulates actin filament networks and enhances motility (Chan et al., 2008, Nogalski et al., 2011). Here, we now show that, subsequent to virus binding and in response to the latency-associated upregulation of HCLS1, latent infection of monocytes results in increased stability of filamentous actin, which, in turn, enhances monocyte migration.

A number of studies have linked the actin filament association of HCLS1 with cell motility of natural killer (NK) cells, DCs, and neutrophils (Dehring et al., 2011, Hao et al., 2005, Latasiewicz et al., 2017, Mukherjee et al., 2015, Uruno et al., 2003). Depletion of HCLS1 from NK cells renders them less motile (Mukherjee et al., 2015). Furthermore, knockout of HCLS1 in the mouse model system decreases neutrophil rolling, adhesion, and migration across the endothelial cell layer. Although it is established that the rolling, adhesion, and migration properties of monocytes, like other leukocytes, help them extravasate across the endothelial cell layer (Martin et al., 2007), it is not known whether HCLS1 plays a role in such monocyte migration and endothelial cell layer transit.

Our analyses now show that latently infected monocytes, in which HCLS1 is profoundly upregulated, have increased motility as well as increased ability to adhere to endothelial cells in a vascular flow system and that they are also more able to cross endothelial cell layers. We confirmed that these effects directly involved latency-associated HCLS1 upregulation by showing that that latently infected cells, in which HCLS1 was knocked down by RNAi, no longer showed latency-associated increases in adherence to vascular layers under flow conditions.

Finally, we also show that mycophenolate mofetil (MMF), which modulates actin polymerization (Chaigne-Delalande et al., 2008, Dubus et al., 2003) and prevents monocyte adhesion and transmigration (Glomsda et al., 2003, Park et al., 2016), also prevented latently infected CD14+ monocytes from adhering to the endothelial layer during physiological flow conditions. This is consistent with the view that it is the increased stability of filamentous actin in latently infected monocytes, mediated by HCLS1 upregulation, which enhances their motility and crossing of endothelial cell layers.

We suggest that the increased endothelial adherence and increased motility of latently infected monocytes, mediated by latency-associated upregulation of HCLS1, could help monocytes latently infected with HCMV to extravasate from blood vessels into surrounding tissues to aid dissemination of the latent monocyte reservoir to tissue sites of reactivation.

## Results

### Analysis of HCMV Latently Infected CD14+ Monocytes Shows a Marked Increase in Cellular Protein HCLS1

**Figure 1:**
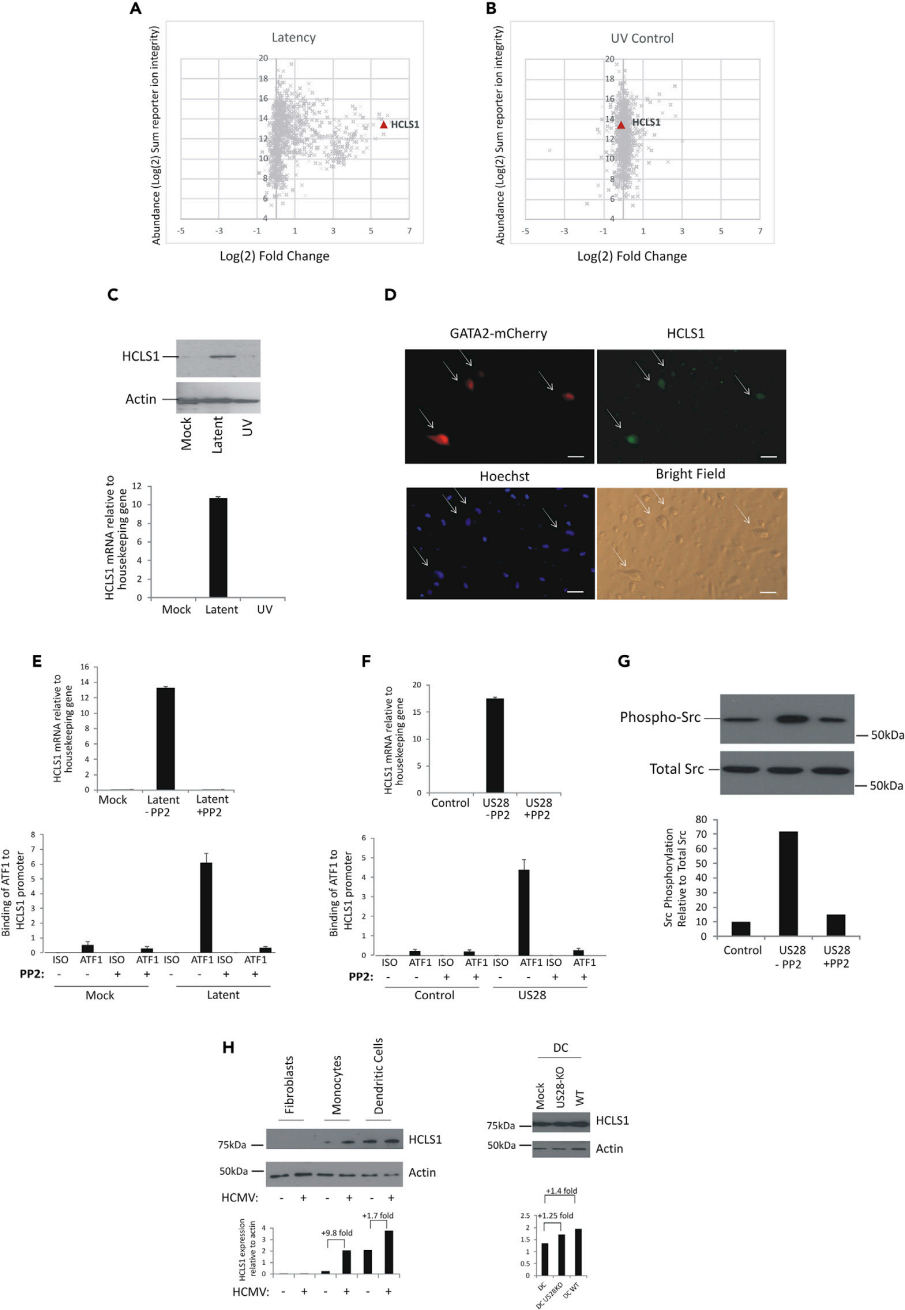
HCLS1 Levels Are Increased during HCMV Latency which Is Regulated by Src Phosphorylation (A–H) CD14+ monocytes were either left uninfected or infected with TB40E-SV40GFP to allow sorting of the latently infected cells on the basis of GFP expression. Cells latently infected for 4 days, or after 4-day infection with UV-inactivated virus, were harvested for total cell proteomics analysis (A and B); western blot analysis or RT-qPCR analysis for HCLS1 and actin (C). Alternatively, CD14+ primary monocytes were infected with TB40E-GATA2mCherry and then co-stained for HCLS1 using primary HCLS1 antibody followed by fluorescein isothiocyanate-conjugated secondary antibody. Red, green, and blue channels as well as bright-field images are shown (scale bar, 30 μM), virally infected cells are highlighted with arrows (D). CD14+ monocytes were also either mock infected or infected with TB40E-SV40GFP and either treated with Src inhibitor PP2 or left untreated. Then they were sorted for GFP expression, and the levels of HCLS1 mRNA were analyzed by RT-qPCR (E, top panel); ATF1 occupancy on the HCLS1 promoter in these cells was determined by chromatin immunoprecipitation assay using either an ATF1-specific antibody or an isotype-matched control antibody (E, bottom panel). In addition, wild-type (WT) and US28-expressing THP-1 cells were also treated or untreated with PP2 and then analyzed for levels of HCL1 RNA by RT-qPCR relative to GAPDH housekeeping gene (F, top panel), and ATF1 occupancy on the HCLS1 promoter was determined using ATF1-specific antibody or isotype-matched control antibody (F, bottom panel). RT-qPCR graphs represent three replicates with standard deviations shown (E and F). Finally, WT THP-1 (empty vector-transduced “control”) cells or THP-1 cells overexpressing US28 (“US28”) were either treated with PP2 or left untreated and analyzed by western blot for phosphorylated Src and actin (as a loading control). Bands were analyzed by densitometry as shown graphically (G). Finally, undifferentiated monocytes, monocytes that had been terminally differentiated into DCs (using granulocyte-macrophage colony-stimulating factor/interleukin-4 and lipopolysaccharide), and fibroblasts were either left uninfected (−) or infected with TB40E-SV40GFP (+) (left panel) or with the Titan strain of HCMV, which expresses a UL32-GFP fusion protein (WT) or the US28 deletion version of the same virus (US28-KO) (right panel). Myeloid cells were then sorted at 4 d post-infection (dpi), based on SV40-GFP expression, and fibroblasts were also sorted at 4 dpi based on GFP-UL32 expression; then all cells were harvested for HCLS1 and actin western blot analysis (H).

Recently, we carried out a total proteomic screen of HCMV latently infected CD14+ monocytes, which we had confirmed were truly latently infected by RNA analysis and virion release assays, and identified proteins that were changed as a result of latent infection (Elder et al., 2019). Further analysis of these data identified that one of the most highly upregulated proteins associated with latent infection was HCLS1 (Figure 1A), which was not upregulated following infection with a UV-inactivated virus (Figure 1B). Therefore, we validated this upregulation of HCLS-1 during latent infection. To do this, we infected CD14+ monocytes with an SV40GFP-tagged TB40E HCMV, which allowed us to enrich for GFP+ latently infected cells, and used western blot analysis to analyze levels of HCLS1. This confirmed that HCLS1 is substantially increased during latent infection at both the protein (western blot, Figure 1C) and mRNA (RT-qPCR, Figure 1C) levels, but not in response to UV-inactivated virus (Figures 1B and 1C). We further analyzed this latency-associated increase in HCLS1 in latently infected cells by indirect immunofluorescence (Figure 1D). To do this, monocytes were latently infected with a second recombinant TB40E isolate expressing mCherry, which allows latently infected cells to be identified for much longer times after infection and overcomes the waning of GFP expression, which is known to occur when using a GFP-tagged virus (Krishna et al., 2016), and then stained these cells for HCLS1. This confirmed that latently infected cells (mCherry positive) had increased expression of HCLS1 (Figure 1D). Taken together these data show that cells latently infected with a number of different virus isolates increase expression of HCLS1 at both the protein and RNA levels.

### Upregulation of HCLS1 during Latent Infection Is Mediated by ATF1 in an Src-Dependent Manner

To try to address the mechanism by which HCLS1 is upregulated during latency we examined the HCLS1 enhancer regions for known activatory transcription factor-binding sites using Genecard analysis and confirmed this using Physbinder, as described in the Transparent Methods. One transcription factor that was predicted to bind to the HCLS1 promoter was ATF1, and this particularly came to our attention because it is known to be upregulated by Src signaling, a signaling pathway known to be involved in infection of monocytes by HCMV (Nogalski et al., 2011) (Shi et al., 2011) (Hokari et al., 2005).

Therefore, we interrogated whether increased levels of HCLS1 in latently infected cells occurred via the phosphorylation of Src and subsequent signaling to allow recruitment of ATF1 to the HCLS1 promoter. To do this, we treated latently infected cells with a known inhibitor of Src signaling, PP2. Figure 1E (top panel) shows that consistent with Src signaling playing a role in the induction of HCLS1 in infected latently infected monocytes, the latency-associated increase in HCLS1 is abrogated by the PP2 Src signaling inhibitor.

We pursued this further by testing whether the latency-associated increase in HCLS1 expression resulted from increased ATF1 binding to the HCLS1 promoter. Figure 1E (bottom panel) shows that significant ATF1 occupancy on the HCLS1 promoter only occurred in latently infected cells and that this was prevented by PP2 Src inhibitor. These data, therefore, suggest that, during latency, HCLS1 is upregulated via Src signaling, which mediates ATF1 binding to the HCLS1 promoter.

Previous work has shown that during HCMV latency, the viral protein US28 is able to induce a number of cellular signaling changes in undifferentiated myeloid cells, quite different to those induced in cells undergoing a lytic infection (Krishna et al., 2017). Interestingly, these changes during latent infection include alterations to Src signaling. Therefore, we tested whether the viral US28 gene product could be involved in HCLS1 upregulation in an Src phosphorylation-dependent manner to increase ATF1 occupancy at the HCLS1 promoter. Figure 1F (top panel) shows that, in myelomonocytic THP-1 cells, overexpression of US28 in isolation results in upregulation of HCLS1 at the RNA level. Furthermore, this US28-mediated upregulation of HCLS1 is inhibited if these cells are treated with the Src inhibitor PP2. Figure 1F (bottom panel) also shows that US28-expressing THP-1 cells have increased ATF1 occupancy on the HCLS1 promoter and that, as with CD14+ cells during HCMV latency, this is also inhibited by PP2.

Finally, Src phosphorylation, itself, was analyzed in the US28-expressing cells by western blot. Figure 1G shows that Src phosphorylation increased after overexpression of US28 in THP-1 cells, and this was inhibited by treatment with Src inhibitor (PP2).

As US28 is also known to target Src phosphorylation during lytic infection (Streblow et al., 1999), we tested whether US28 could also mediate HCLS1 upregulation during a lytic infection. To do this, two cell types that support lytic infection, fibroblasts and DCs, were infected alongside monocytes for 4 days and analyzed for HCLS1 and actin content by western blot.

First, we were unable to detect HCLS1 expression in untreated fibroblasts consistent with the known low levels of HCLS1 in this cell type (Shin et al., 2012), and their lytic infection with HCMV failed to show any induction of HCLS1 (Figure 1H, right panels).

As before (Figure 1), latent infection of monocytes showed an approximate 10-fold increase in HCLS1 (Figure 1H, left panels). In contrast, lytic infection of monocyte-derived DCs resulted in less than a 2-fold increase in HCLS1, and this was above an already high background level of HCLS1 (Figure 1H, left panels). To test whether this minor increase in HCLS1 during lytic infection was US28 dependent, we repeated this analysis using a US28 deletion virus but still observed an increase in HCLS1 expression in infected DCs (Figure 1H, right panels).

Consequently, lytic infection of DCs results in only a small increase in HCLS1 over an already high basal level of HCLS1 expression in these cells, and this small increase is only partially US28 dependent.

Taken together, these data suggest that latent infection of monocytes results in a substantial increase in HCLS1, which is, at least in part, mediated by US28. In contrast, lytic infection of monocyte-derived DCs showed only a minor increase in HCLS1 over already high background levels of HCLS1 and argues that the most profound functional effects of HCLS1 induction are likely to be during latent infection.

### HCMV Latent Infection Results in Monocytes with Stabilized F-actin

We next addressed the potential role of HCLS1 induction during latent infection. As a cortactin homolog, HCLS1 is known to stabilize F-actin and increase cell motility (Dehring et al., 2011, Martin et al., 2007, Uruno et al., 2003). Consequently, we reasoned that the increased expression of HCLS1 during latency could prevent destabilization of F-actin, which can result from cytochalasin D (CD) treatment (Aunis and Bader, 1988, Sontag et al., 1988, Thieler et al., 1995).

**Figure 2:**
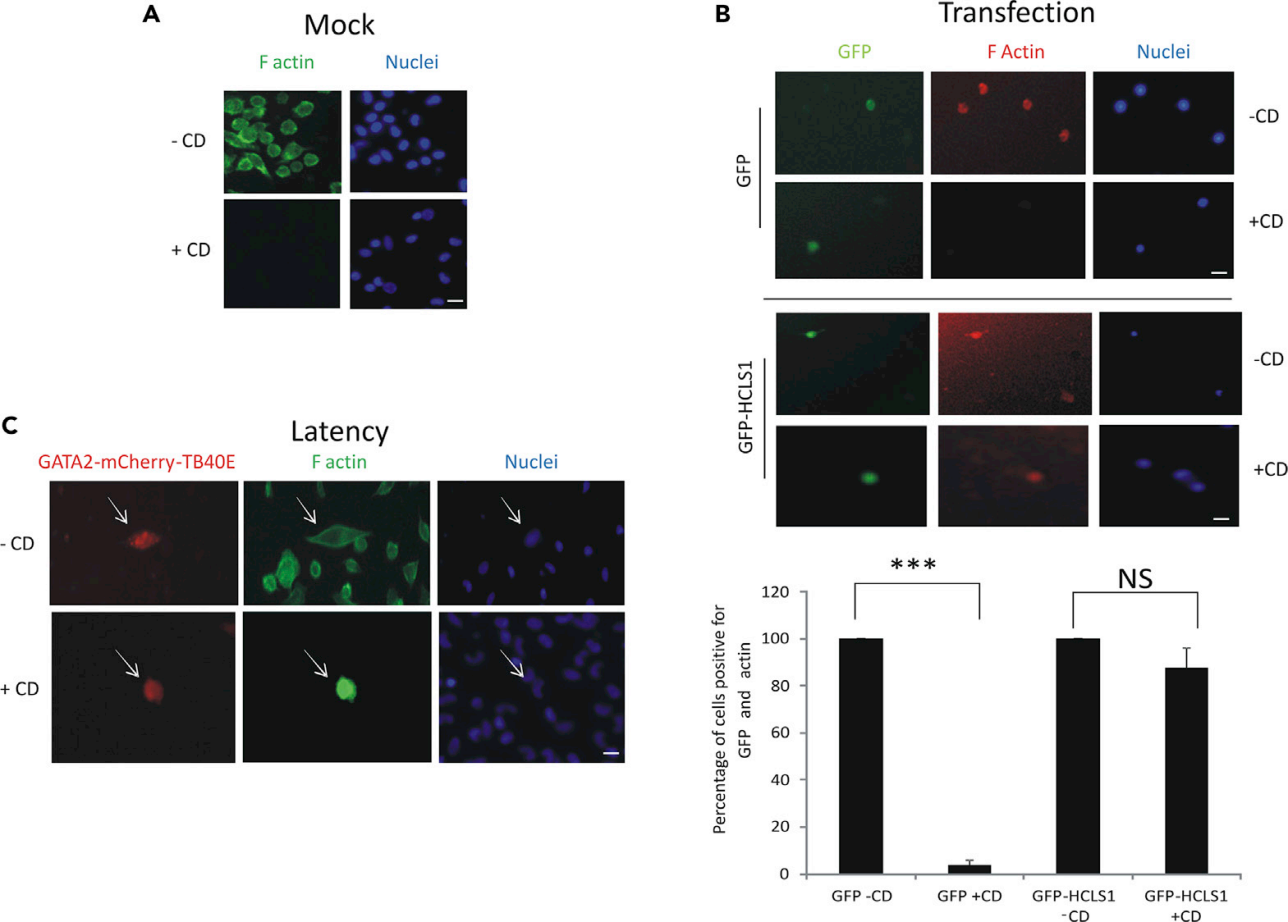
HCLS1 Upregulation during Latency Leads to Increased and Stable Actin Filament Formation (A–C) (A) CD14+ monocytes in which actin filament formation had been disrupted using CD were analyzed by fluorescence for F-actin (green) and Hoechst (blue). Alternatively, CD14+ monocytes overexpressing GFP or GFP-HCLS1 were analyzed for actin filament formation in the presence and absence of CD and analyzed by fluorescence. GFP (green), actin (red), and Hoechst (blue) stains are shown with cells enumerated in three experiments of triplicate wells. Percentage of cells that are both GFP and actin positive are represented; ***p < 0.0001; NS, not significant (B). Finally, actin filament formation in the presence of HCMV latency was assessed in the presence and absence of CD. CD14+ monocytes were infected with TB40E-GATA2mCherry virus, which expresses mCherry under the control of the GATA2 promoter (red), and stained for actin filaments (green) and Hoechst (blue) and analyzed by fluorescence, the virally infected cell is highlighted with arrows (C) (scale bars, 30 μm).

Figure 2A shows that, treatment of control untreated monocytes with CD resulted in a substantial decrease in detectable F-actin (CD+). In contrast, overexpression of HCLS1 in isolation in monocytes was able to prevent this F-actin disruption by CD treatment (Figure 2B), which has only been shown for fully differentiated DCs previously (Dehring et al., 2011). We next tested whether this protective effect of increased HCLS1 on CD-mediated destabilization of F-actin also occurred in latently infected monocytes. Figure 2C shows that uninfected monocytes (mCherry negative) treated with CD lost F-actin staining. In contrast, this CD-mediated decrease in F-actin staining was not observed in latently infected (mCherry positive) monocytes (Figure 2C).

Taken together, these results show that the upregulation of HCLS1 during HCMV latency results in increased stabilization of F-actin structure.

### Overexpression of HCLS1 as well as Latent Infection of CD14+ Monocytes Leads to Increased Cell Motility

**Figure 3:**
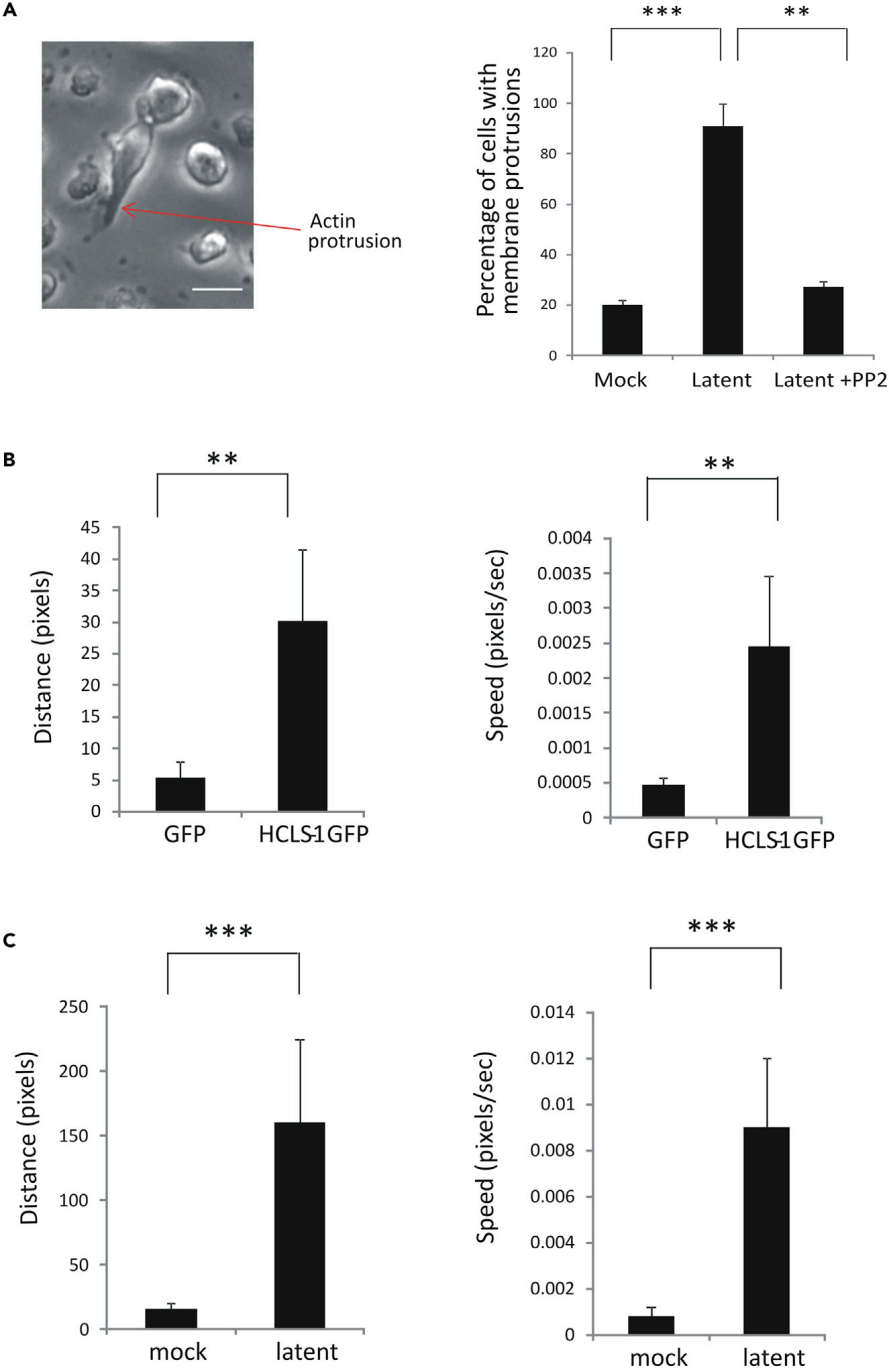
Monocytes Overexpressing HCLS1 Are More Motile, and This Is Recapitulated after Latent Infection (A–C) CD14+ cells were latently infected for 4 days with HCMV and then analyzed for membranous protrusions by phase contrast microscopy and cell counting (scale bar, 30 μm) (A). The frequency of membranous protrusions during HCMV latency compared with mock infected cells or latently infected cells that have been treated with PP2 1 h post infection are shown graphically where data represent three experiments with standard deviation; *** p < 0.0001, **p < 0.001 (A). CD14+ monocytes were transfected with either GFP or HCLS1-GFP, and then Cellomics analysis was used to assess distance moved and speed of movement of the cells (B). Alternatively, CD14+ monocytes were either left uninfected or infected with the TB40E-GATA2mCherry HCMV virus (to allow visualization of latent cells by red fluorescence) and latency established for 4 days before they were analyzed using Cellomics for cell motility by assessing distance moved and their speed of movement (C). Data represent triplicate experiments, and motility was analyzed for 100 cells per sample. Standard deviation error bars and significance determined using the Student's t test; **p < 0.001, ***p < 0.0001 (B and C).

One consequence of increased HCLS1, and subsequent stabilized F-actin can be increased motility of cells in certain cell types (Dehring et al., 2011, Mukherjee et al., 2015). Therefore, we overexpressed HCLS1 by electroporation with HCLS1-GFP or GFP expression plasmids into monocytes, a cell type in which HCMV latency resides, and analyzed the frequency of actin protrusions, as a surrogate for motility, as determined by cells that phenotypically contained protrusions that stained for F-actin (Melak et al., 2017). Initially, we analyzed latently infected cells for the presence of actin protrusions. Figure 3A clearly shows that latently infected cells had substantial increase in actin protrusions (an example of an actin protrusion is highlighted in phase contrast, top panel, Figure 3A), suggesting increased motility, yet the monocyte protrusions were inhibited if the cells were treated with PP2, a drug known to affect actin-based motility (Figure 3A right panel). To test this directly, these HCLS1-GFP-positive cells were also analyzed by Cellomics time-lapse imaging at 24 h post electroporation. Figure 3B shows that cells that had been electroporated with HCLS1-GFP were substantially more motile and moved further than those transfected with GFP alone. Still images and videos of these analyses are also shown in Figure S1B. Therefore, monocytes, like other cell types, also increase their motility in response to overexpression of HCLS1.

Next, to test whether latently infected monocytes also have increased motility, we again used Cellomics time-lapse live cell imaging to assess cell movement in latently infected populations of CD14+ monocytes. We employed TB40E-GATA2-mCherry virus for these analyses to allow long-term tracking of latently infected cells. Figure 3C shows the quantification of movement and distance traveled by latently infected monocytes compared with uninfected bystander cells in the same population. Latently infected CD14+ monocytes appeared more pleomorphic (Figure 3A) and were substantially more motile over the time course studied than uninfected bystander cells. Still images and videos of these analyses are shown in Figure S1 and Videos S1, S2, and S3.

Video S1. Latent Cell Moving, Related to Figure S1A

Video S2. GFP, Related to Figure S1B

Video S3. HCLS1, Related to Figure S1B

These data argue that latent infection with HCMV enhances the motility of CD14+ monocytes.

### Latently Infected CD14+ Monocytes Adhere to Endothelial Cells More Efficiently and Are More Able to Cross Endothelial Cell Layers

HCLS1 expression is also known to mediate neutrophil rolling, adhesion, and migration across the endothelium. Similarly, the rolling, adhesion, and migration properties of monocytes also help them extravasate across endothelial cell layers (Martin et al., 2007). Consequently, we reasoned that latency-associated HCLS1 increases in monocytes might affect monocyte motility, adhesion, and transmigration across endothelial cell monolayers.

**Figure 4:**
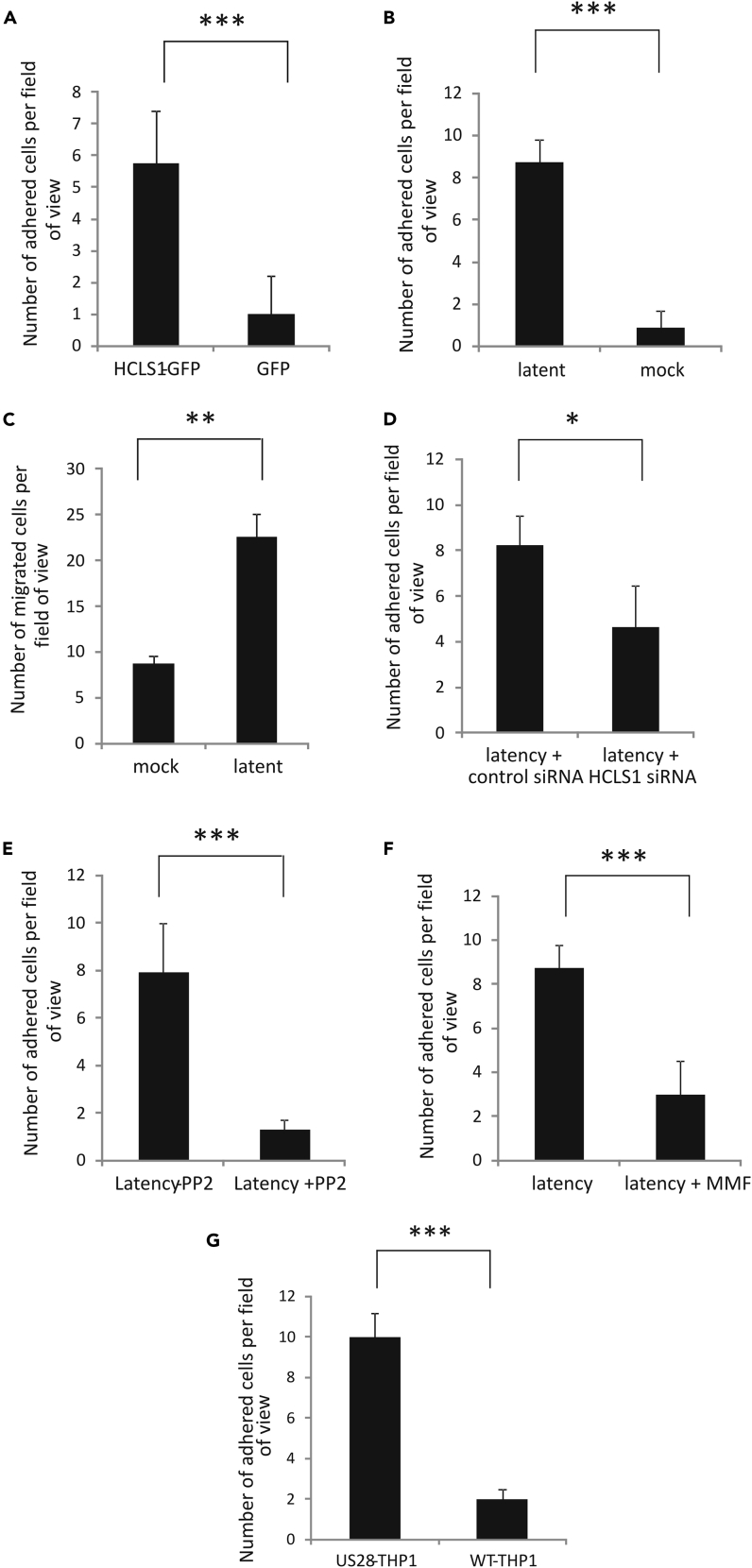
Monocytes Latently Infected with HCMV Show Enhanced Adhesion to, and Transmigration through, Endothelial Cell Layers in an HCLS1-Dependent and Src Phosphorylation-Dependent Manner (A–G) CD14+ cells that had been transfected with either HCLS1-GFP or GFP (A) or that had been left uninfected (mock) or infected with TB40E-GATA2mCherry (B) were assessed for their ability to adhere to endothelial cell layers. CD14+ cells that had been either mock infected or infected with TB40-GATA2mCherry virus were also assessed for their ability to migrate across endothelial cell layers in transwell assays (C). CD14+ cells that had been infected with TB40E-GATA2mCherry were treated with either scramble siRNA or siRNA specific to HCLS1 and similarly assessed for their ability to adhere to the endothelial cell layer (D). CD14+ cells that had been latently infected with TB40E-GATA2mCherry were also assessed for the ability to adhere to the endothelial cell layer in the presence of either Src inhibitor PP2 (E) or the drug MMF (F). Finally, “WT-THP1” (empty vector transduced) cells or THP1 cells expressing US28 “US28-THP1” were assessed for the ability to adhere to the endothelial cell layer (G). Experiments were carried out in replicate, and data represent triplicate experiments and show standard deviation error bars and significance determined using the Student's t test; *p < 0.01, **p < 0.001, ***p < 0.0001.

To test this, we first analyzed whether monocytes overexpressing HCLS1 were more able to adhere to endothelial cells under *in vitro* flow simulation. In this system (Butler et al., 2011, Zhao et al., 2016), endothelial cells were allowed to adhere to an artificial vessel and monocytes overexpressing GFP or HCLS1-GFP were perfused through the vessel at a physiological flow rate of 0.4 mm/mL and then fixed and analyzed by fluorescence. Figure 4A clearly shows that monocytes overexpressing HCLS1 showed increased adherence to the endothelial cell layer under physiological flow conditions (the electroporation efficiency for these plasmids was between 40% and 80% based on GFP expression as shown in Figure S2A). Therefore, as with neutrophils, increased expression of HCLS1 in monocytes can also mediate adherence to endothelial cell layers.

We then repeated these analyses with monocytes that had been not only latently infected with GATA2-mCherry virus but also co-stained with calcein (to allow differential detection of bystander monocytes and latently infected monocytes). Figure 4B shows that latently infected monocytes also adhered to the endothelial layer under flow conditions more efficiently than uninfected monocytes.

Next, we addressed whether latently infected monocytes were more adept at crossing endothelial cell layers. To do this, we sorted mCherry-positive latently infected monocytes and assessed their ability to cross a transwell membrane, which had been overlaid with endothelial cells. Figure 4C shows that latently infected monocytes were more efficient at crossing these endothelial-coated transwell membranes than uninfected monocytes.

Our data, so far, clearly showed that overexpression of HCLS1 in monocytes increased their motility and adhesion to endothelial cells as well as their ability to cross endothelial cell layers under flow conditions and that latently infected cells showed similar enhanced capabilities. However, this did not provide direct evidence that it was the latency-associated increase in HCLS1 that was mediating these effects in latently infected monocytes. To address this directly, we repeated the analysis of the ability of latently infected monocytes to adhere to endothelial monolayers, but in monocytes in which we had ablated the latency-associated upregulation of HCLS1 by RNA interference.

To do this, CD14+ monocytes were infected with HCMV and, subsequently, HCLS1 expression was inhibited by electroporation of these latently infected cells with HCLS1-specific small interfering RNA (siRNA). This resulted in good levels of knockdown of HCLS1 protein (Figure S2B). Figure 4D shows that knockdown of HCLS1 in latently infected CD14+ monocytes resulted in a significant reduction in their ability to adhere to endothelial cell layers under physiological flow conditions compared with those electroporated with a scramble control siRNA. To further validate the role of HCLS1 in these effects we also tested whether inhibition of Src, which we showed in Figure 1 to be important for HCLS1 induction in latently infected cells, had any effect on the ability of monocytes to adhere to endothelial cell layers under physiological flow conditions. Adherence of latently infected monocytes was decreased when Src phosphorylation was inhibited (Figure 4E), demonstrating that Src signaling was indeed playing a role in the HCLS1-mediated motility observed during latent infection.

MMF, besides a general immune suppressant, is also known to modulate actin polymerization (Chaigne-Delalande et al., 2008), thereby blocking monocytes from transmigrating across endothelial cell layers (Juttner et al., 2009). Consequently, we reasoned that if increased adherence and transendothelial migration of latently infected monocytes were due to latency-associated stabilization of F-actin through HCLS1 upregulation, MMF should inhibit adhesion of latently infected monocytes to endothelial cells. To test this, latently infected CD14+ monocytes were pre-incubated with MMF before perfusion over endothelial cell layers. Figure 4F shows that, following treatment with MMF, latently infected CD14+ monocytes were significantly less able to adhere to the endothelial cell layer.

Finally, on the basis of the fact that our data showed that US28-mediated activation of Src drives overexpression HCLS1 and subsequent HCLS1-mediated increase in endothelial cell adherence, we tested whether monocytic cells overexpressing US28 had increased endothelial cell adherence. Figure 4G shows that THP1 cells that overexpress US28 are, indeed, more able to adhere to endothelial cell layers.

Taken together, these data argue that latent infection of CD14+ monocytes results in increased levels of cellular HCLS1, which stabilizes actin filaments and concomitantly results in enhanced monocyte motility. This, in turn, aids monocyte adhesion to, and transmigration through, the endothelial cell layer. We believe that such effects could play an important role in virus dissemination from the peripheral blood compartment to tissue sites of reactivation.

## Discussion

The work presented here shows that the latency-associated upregulation of HCLS1, which we previously identified in an unbiased analysis of changes in the cell proteome upon HCMV latent infection (Elder et al., 2019), modulates cell motility of latently infected cells and increases their ability to extravasate across endothelial cell layers.

HCLS1 is a substrate of antigen receptor-coupled tyrosine kinases, and it is only expressed in tissues and cells of hematopoietic origin (Yamanashi et al., 1993). In myeloid cells, HCLS1 has a number of established functions including the regulation lymphocyte trafficking (Scielzo et al., 2010), neutrophil chemotaxis (Cavnar et al., 2012), and growth arrest and apoptosis (Fukuda et al., 1995). Importantly, it also binds F-actin and can increase the rate of actin assembly (Hao et al., 2005, Uruno et al., 2003). Interestingly, such changes in actin assembly are known to be important for cell motility of NK cells, DCs, and neutrophils as well as neutrophil rolling, adhesion, and migration across the endothelial cell layer (Dehring et al., 2011, Hao et al., 2005, Latasiewicz et al., 2017, Mukherjee et al., 2015, Uruno et al., 2003).

Our analyses also show that the mechanism by which HCLS1 is upregulated during latency is by increasing ATF1 association with the HCLS1 promoter by Src signaling and is consistent with previous observations in other conditions (Hokari et al., 2005, Shi et al., 2011). This is particularly pertinent because HCMV lytic infection is known to regulate Src signaling (Streblow et al., 1999) and this upregulation during lytic infection is mediated by the viral US28 gene product. Importantly, US28 is also a latency-associated viral gene product (Bego et al., 2005, Poole et al., 2013), which has been suggested to increase Src phosphorylation in latently infected cells (Krishna et al., 2017). In addition, Src signaling is also known to increase upon monocyte binding and entry of HCMV (Nogalski et al., 2011), a known site of HCMV latency. Therefore, not only does initial binding of HCMV to monocytes alter Src phosphorylation, which then modulates actin filament formation via paxillin regulation (Nogalski et al., 2011), but also this is continued, once latency has been fully established, via regulation of HCLS1. As Src phosphorylation has also been published as an effect of US28 during lytic infection, we also addressed whether US28 can mediate HCLS1 upregulation during lytic infection. We found that, in contrast to latent infection, lytic infection of DCs resulted in only minor increases in HCLS1, over an already high basal level of HCLS1 expression and that US28 likely played only a minor role in this upregulation. Consequently, we believe that the most profound functional effects of HCLS1 induction are likely to be during latent infection.

Consistent with the known effects of HCLS1 in regulating F-actin filaments, which can lead to enhanced motility, latently infected monocytes have more stable actin and are more mobile. One consequence of increased actin filaments and enhanced motility in monocytes is an increased ability to adhere to, and cross, the endothelial cell layer. Again, consistent with this, latently infected cells showed enhanced adherence to endothelial cell layers and increased ability to migrate across them. In all cases, our analyses showed that it was increased HCLS1 that mediated these latency-associated changes in monocyte motility, adherence, and trafficking as inhibition of the latency-associated increase in HCLS1 by RNAi prevented them.

One obvious advantage of these effects on monocytes during latent infection could be in viral dissemination in the myeloid lineage. *In vivo*, latent HCMV can be carried in peripheral blood monocytes (Reeves et al., 2005b, Taylor-Wiedeman et al., 1991). Consequently, any increased ability of a latently infected cell in the peripheral blood to adhere to and cross the endothelial cell layer would very likely enhance dissemination of the latent virus to tissue sites in the host conducive to monocyte differentiation and subsequent reactivation. HCMV is not the only herpes virus to increase endothelial cell motility during latency. The gamma herpesviruses Epstein-Barr virus (Tugizov et al., 2007) and Kaposi's sarcoma virus also promote endothelial transmigration following latent infection of endothelial cells (DiMaio et al., 2011, Giffin et al., 2015), although, in contrast to HCMV, lytic infection of the endothelial layer prevents neutrophil migration (Butler et al., 2011). Similarly, HIV-1, which can establish latency in resting CD4+ cells, promotes endothelial transmigration of infected monocytes (He et al., 2016, Maslin et al., 2005, Noguchi et al., 2007, Westhorpe et al., 2009), suggesting that this may be a common feature of blood-borne viruses, which establish latent life cycles.

In conclusion, we believe that changes in myeloid-specific HCLS1 imparted by latent infection of monocytes with HCMV can alter their adhesion to endothelial cells and promote transendothelial migration and that this could aid efficient dissemination of HCMV latently infected cells from the peripheral blood to potential tissue sites of reactivation.

### Limitations of the Study

As with all studies on species-specific pathogens, the inherent lack of an animal model is a limitation to any study. However, with increasingly more effective experimental latency models for the study of HCMV these types of studies are becoming more informative and directly relevant to the *in vivo* system.

## Methods

All methods can be found in the accompanying Transparent Methods supplemental file.

## Supplemental Information

Document S1. Transparent Methods and Figures S1–S3
