# Supplementary material for: Human Cytomegalovirus Upregulates Expression of HCLS1 Resulting in Increased Cell Motility and Transendothelial Migration during Latency
Source: iScience. 2019 Sep 14;20:60–72. doi: 10.1016/j.isci.2019.09.016 (PMC6817630; doi:10.1016/j.isci.2019.09.016)
Supplement: Document S1. Transparent Methods and Figures S1–S3 [file mmc1.pdf]

ISCI, Volume *20*

Supplemental Information

Human Cytomegalovirus Upregulates Expression of HCLS1 Resulting in Increased Cell Motility and Transendothelial Migration during Latency

Yusuf Aslam, James Williamson, Veronika Romashova, Elizabeth Elder, Benjamin Krishna, Mark Wills, Paul Lehner, John Sinclair, and Emma Poole

Supplementary figures and methods

Supplementary Figure Legends

Supplementary figure 1 related to figure 3

A) CD14+ monocytes were infected with TB40E-GATA2mCherry and latency established for 4 days before analysing by cellomics over a period of 18h, as shown. Video images were also acquired of the cells over this period (A). Alternatively, monocytes were electroporated with a plasmid expressing GFP or HCLS1-GFP for 24h before analysing by cellomics over a period of 18h as shown. Videos were also taken of the cells over this time period (B).

Supplementary figure 2 related to figure 4

Monocytes were electroporated with plasmids expressing GFP, HCLS1-GFP or an siRNA conjugated to FAM (for visualisation to assess transfection efficiency) and then analysed for green fluorescence 24h later (A). Cells which had been electroporated with either a control siRNA or an siRNA to HCLS1 were harvested for the western blot of actin and HCLS1 proteins (B).

Supplementary figure 3 relating to figures 1, 2, 3 and 4

HCMV latency validation. Primary CD14+ monocytes were either uninfected or infected with HCMV for 4 days before analysing by RT-qPCR (A) and then either leaving undifferentiated or differentiating into DCs using GM-CSF/IL-4. After this time supernatants were taken and transferred onto indicator fibroblasts to assess for virus release by infectious foci detection (B).

Supplementary figures

Supplementary movies are in a separate file

Supplementary figure 1

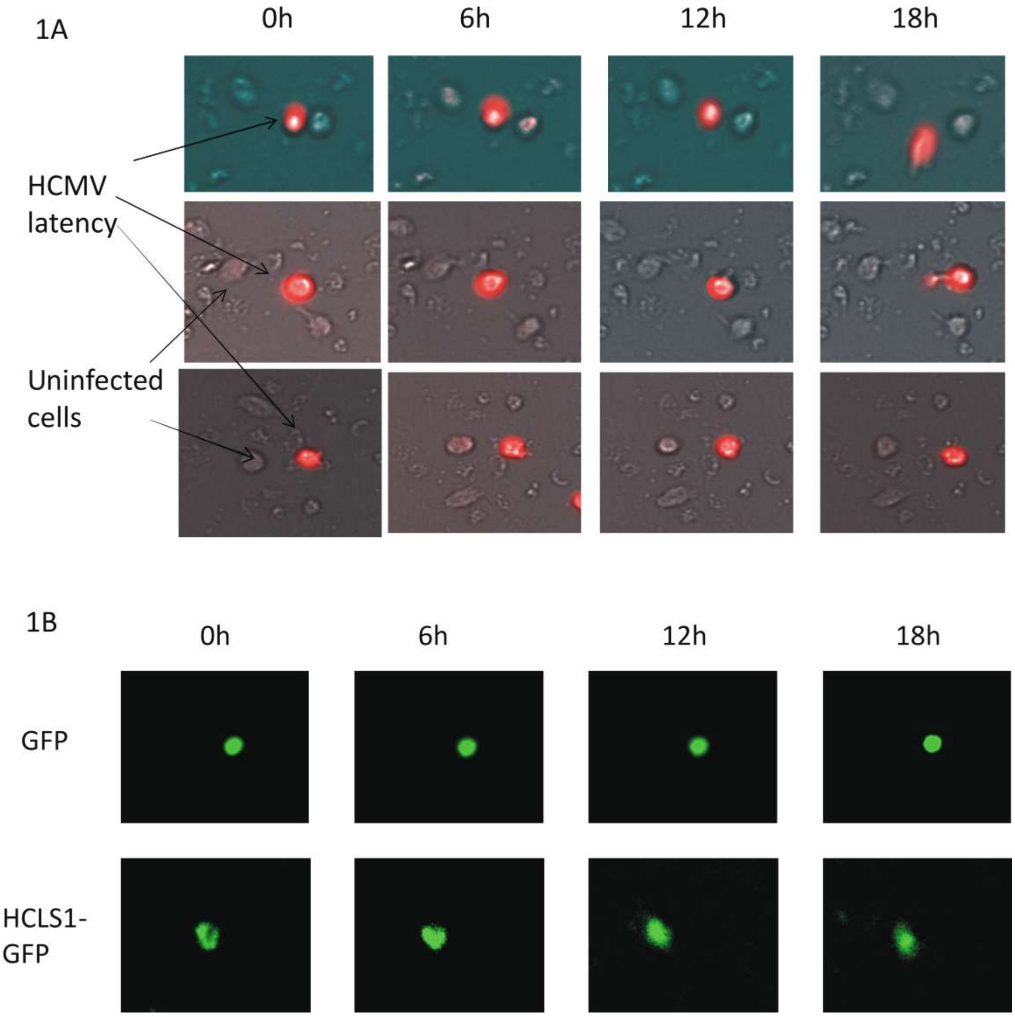

Supplementary figure 2

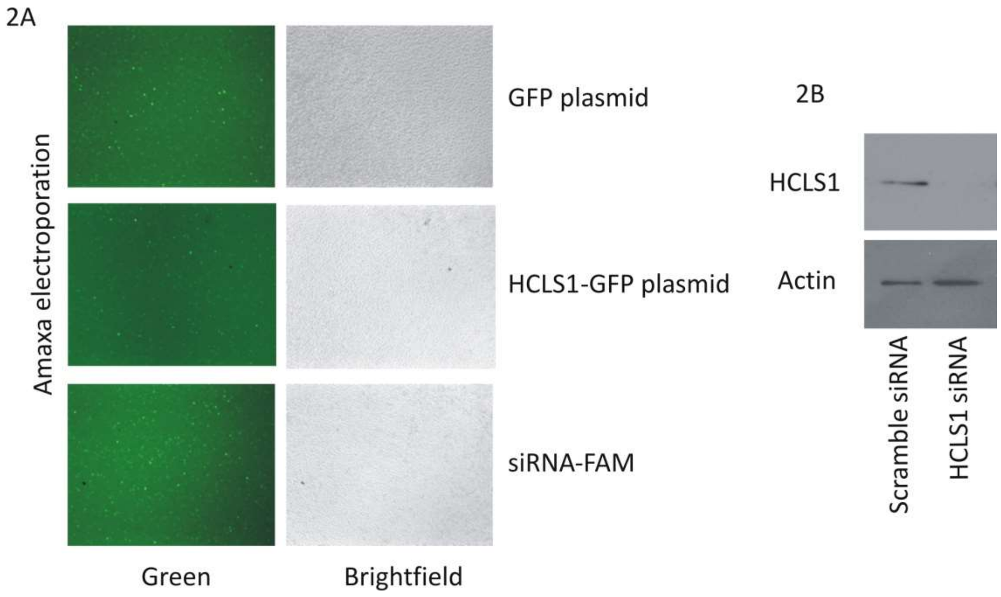

Supplementary figure 3

3A

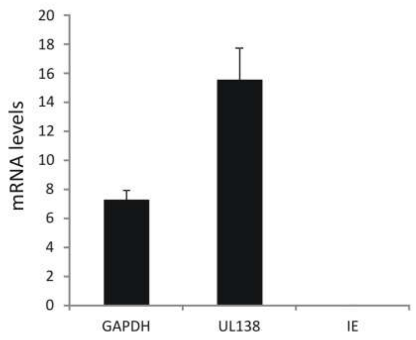

3B

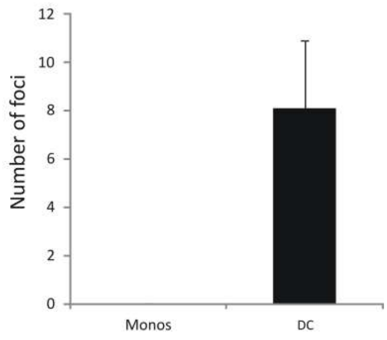

Transparent Methods

Cells and viruses

Primary monocytes were isolated from apheresis cones or venous blood as described previously (Mason et al., 2012). HAEC (Promcell) and HUVEC (ATCC) were cultured as described by the suppliers. Human Foreskin Foetal Fibroblasts (HFFFs) were obtained from ATCC and cultured as described previously (Poole et al., 2006).

TB40E-SV40-GFP and TB40E-GATA2mCherry viruses have been described previously (Elder, 2019; Krishna et al., 2016).

Titan WT and Titan US28 deletion virus have been described previously (Krishna et al., 2017a)

Ethical Statement

All research describing studies on primary human material with HCMV were assessed and approved by the Cambridge Local Research Ethics committee. Informed consent was received from blood donors with the Cambridge Local Research Ethics committee and the Cambridge Internal Review Board. Cells were harvested from healthy adult donors, and the decision to use tissue was not affected by gender and age, as this was not important to the studies performed.

Generation of CTCF shRNA and US28-expressing cell lines by lentiviral transduction

pHRsinGKpuro and pHRsinUbEm lentiviral expression vectors were a kind gift from Dr D van den Boomen, University of Cambridge. The sequence encoding US28 was cloned into pHRsinUbEm using the BamHI and NotI sites. The lentiviral expression vector encoding CTCFshRNA and puromycin resistance (Santa Cruz Biotechnology).

To generate lentiviral particles, 293T cells were seeded into 6-well plates at $5x10^5$ cells/well. Approximately 6 hours later, 1250 ng of lentiviral expression vector, 625 ng of lentiviral packaging vector psPAX and 625 ng of lentiviral envelope vector pMD.2G (both gifts from S. Karniely, Weizmann Institute, Israel) were transfected into 293T cells using transfection reagent FuGene6 (Promega) according to the manufacturer's instructions. 24h post transduction, media was replaced with 2.5mL RPMI supplemented with 30% fetal calf serum. 24h post media change, $2.5x10^5$ THP-1 cells were pelleted, and then resuspended in the lentiviral or control supernatant in a 6-well plate. Polybrene was added to the cells at 2 µg/mL and the cells were then centrifuged in the plate at 600x*g* for 45 minutes, and incubated overnight at 37°C/5% CO_2. 5 days post transduction the transduced THP-1 cells were pelleted and resuspended in fresh RPMI supplemented with 10% fetal calf serum. 7 days post transduction, puromycin (Sigma) was added at 2 µg/mLand the selective media was refreshed every 2 days GFP positive cells were sorted by FACS.

In silico transcription factor binding analysis

The HCLS1 enhancer binding sequence was obtained from Genecards https://www.genecards.org/cgi-bin/carddisp.pl?gene=HCLS1 and the identified transcription factor, ATF1, binding site was confirmed using Physbinder http://bioit.dmbr.ugent.be/physbinder/

HCMV latent infection

To establish latency, primary monocytes or a monocytic cell line were infected for 3h with virus at a predicted MOI of 1 and then left for a minimum of 4 days before analysing for latent infection. This was determined by RTqPCR for the presence of the latency associated gene UL138 in the relative absence of the lytic gene IE. Additionally, supernatants were assessed for virus release pre- and post-differentiation into dendritic cells using GM-CSF and IL-4 as previously described (Poole et al., 2014a). An example analysis is shown in supplementary figure 3. We define latency as the presence of latency associated transcripts such as UL138 in the relative absence of the lytic gene IE (Supplementary figure 3A). Crucially, during latency there is no virion production as measured by co-culture of latently infected cell/cell supernatants on fully permissive indicator fibroblasts (Supplementary figure 3B). Latently infected cells used in all our experiments were consistent with this definition of latency.

HCLS1 RT-qPCR

HCLS1 and GAPDH commercial primers were obtained from Qiagen and then RNA was isolated from the indicated samples using the Qiagen RNA isolation kit followed by SYBR green RT-qPCR analysis using standard parameters (Qiagen).

Plasmids, siRNAs and transfection

Plasmids and siRNAs were electroporated into primary monocytes using the Amaxa monocyte transfection kit (Lonza). GFP plasmid was obtained from Lonza whereas HCLS1-GFP plasmid and siRNA scramble and siRNA-HCLS1 were obtained from OriGene and FAM labelled control siRNA from Thermofisher. 1ug of each plasmid or 50nM of each siRNA was electroporated into $3x10^6$ monocytes by Amaxa.

Western blotting, and immunofluorescence Western blotting and immunofluorescence were carried out as described previously (Poole et al., 2012; Poole et al., 2014b) . HCLS1 primary antibody was used for both western blotting and immunofluorescence (rabbit polyclonal, abcam). Actin antibody for western blotting was obtained from Abcam (rabbit polyclonal) both as directed by the manufacturer. Western blots were analysed using autoradiographic film and immunofluorescence computerized image analysis software (Image ProPlus and Image J).The immuofluorescence method has been described previously (Poole et al., 2006) .

Actin filament analysis and drug inductions

To assess actin filament formation, cells were first fixed and then stained with phalloidin which is specific for F-actin using a previously described method (Melak et al., 2017). CD14+ monocytes in

which latency had been established for 6 days with either GATA2mCherryTB40E were either fixed and stained directly with the F-actin specific marker phalloidin 488 or phalloidin 594 (CytoPainter, Abcam) respectively, in the presence of Hoechst. Alternatively, cells were treated with 200nM cytochalasin D (a concentration that is reversible for actin filament formation; (Domnina et al., 1982)) for 1h before washing, staining and visualising by fluorescence microscopy using computerized image analysis software (Image ProPlus and Image J). MMF and PP2 were obtained from Sigma and used at the recommended concentrations from the supplier and as described previously (Melak et al., 2017) (Chan et al., 2012). Specifically, cells were treated with 1uM PP2 1h post and during the establishment of HCMV latency. The media was changed every 24h with fresh PP2 added to the media.

Chromatin Immunoprecipitation (ChIP) The HCLS1 promoter was analysed by ChIP using primers to the enhancer: Forward -TAAAAAGAAACTCACCCCTA and Reverse - AGATTAGGAAAGGCTAGGTC using the Imprint ChIP kit (Sigma) following the manufacturer instructions.

Endothelial migration

Monocyte to transmigration across the endothelial layer was analysed as previously described (Bentz et al., 2006). In brief, endothelial cells were seeded onto the filters of transwell plates before carrying out standard monocyte transwell analysis (Mason et al., 2012). The CD14+ monocytes in which HCMV latency had been established were isolated by live cell sorting (Krishna et al., 2017b; Lau et al., 2016). $1x10^4$ infected or uninfected cells were added to each transwell and after 2 hours the monocytes which had migrated through the endothelial cell layer were enumerated by bright field microscopy using computerized image analysis software (Image ProPlus and Image J).

Endothelial Flow analysis

Adhesion of monocytes to the endothelial cell layer was determined by flow analysis using previously published methods (Butler et al., 2011; Zhao et al., 2016). In brief, endothelial cells were seeded into Ibidi slides $VI^{0.4}$. When the cells reached confluency, $1x10^6$ monocytes were perfused over the endothelial cell layer, within a Perspex chamber maintained at 37degrees, at the physiological flow rate of 0.4ml/min. The flow rate of 0.4 mL/min was pump-controlled. The equivalent shear stress ($\boldsymbol{\tau}$) exerted on the Ibidi $\boldsymbol{\mu}$-Slide $VI^{0.4}$ slide surface at a flow rate of 0.4 mL/min (Φ) was 0.7 dyn/cm^2, calculated from the equation "$\boldsymbol{\tau} = \boldsymbol{\eta}176.1\Phi$". $\boldsymbol{\eta}$ (dynamical viscosity) was

0.01 dyn·s/cm^2. CD14+ monocytes were then washed in PBS before fixing in 4% PFA in PBS and visualising by fluorescence using computerized image analysis software (Image ProPlus and Image J).

Proteomic screen

Lysis, digestion and clean-up of protein preps

From 6 well plates, cells were washed 2x with cold PBS before scraping cells into 200uL 8Murea/50mM TEAB pH 8.5. Samples were quantified by BCA assay and 28ug of each sample was taken and adjusted to the same volume with lysis buffer. Reduction and alkylation was achieved by addition of 10mM TCEP and 20mM iodoacetamide for 20mins at room temperature in the dark followed by quenching with 10mM DTT. 3ug of rLysC (Promega) was added to each sample and incubated at 30 degrees for 3h before diluting the 1:5 with 50mM TEAB and addition of 3ug trypsin (Proteomics Grade, Thermo Fisher Scientific) and incubation at 37 degrees overnight. Samples were acidified by adding 1 volume of 0.2% TFA and formic acid until pH was ~>2. Samples were desalted using in-house prepared microcolumns consisting of Oligo R3 resin (SCIEX) packed behind a C18 plug (Empore, 3M) in p200 pipette tips. Columns were washed with 100uL ACN and equilibrated with 2x100uL 0.1% TFA before loading of samples (3 passes over the column). Columsn were washed with 3x 50uL 0.1% TFA before sequential elution with 20uL each of 40% ACN, 70% ACN and 70% ACN with 1% FA. Samples were dried in a vacuum centrifuge.

TMT Labelling

Samples were resuspended in 20uL 100mM TEAB and to each tube 0.2ug of a unique TMT label for each sample was added in 8.5µL acetonitrile and incubated for 1h at room temperature. Labels were as follows: TMT reactions were quenched by addition of 3µL of 200mM ammonium formate, pooled and dried in a vacuum centrifuge. The sample was then Resuspended in 800µL 0.1% TFA and acidified to ~pH2 with formic acid before performing a C18-SPE cleanup using a Sep-Pak cartridge (Waters) attached to a vacuum manifold. C18 Eluate was dried in a vacuum centrifuge and resuspended in 40µL 200mM ammonium formate, pH10.

High pH Revered Phase Fractionation

Sample was injected onto an Ultimate 3000 RSLC UHPLC system (Thermo Fisher Scientific) equipped with a 2.1 i.d x25cm, 1.7µm particle Kinetix Evo C18 column (Phenomenex). Mobile phase consisted of A: 3% ACN, B:ACN and C: 200mM ammonium formate pH 10. Isocratic conditions were 90% A/10%C and C was maintained at 10% throughout the gradient elution. Separations were carried out at 45 degrees. After loading at 200µL/min for 5 mins and ramping the flow rate to 400µL/min over

5mins the gradient elution proceed as follows: 0-19% B over 10 minutes (curve 3), 19-34%B over 14.25mins (curve 5), 34-50%B over 8.75mins (curve 5), followed by a 10 min wash at 90% B. UV absorbance was monitored at 280nm and 15s fractions were collected into 96 well microplates using the integrated fraction collector. Peptide containing fractions were then orthogonally recombined into 12 fractions and dried in a vacuum centrifuge and resuspended in 10µL 5% DMSO 0.5% TFA for analysis.

LC-MS analysis

All samples were injected onto an Ultimate 3000 RSLC nano UHPLC equipped with a 300µm i.d. x 5mm Acclaim PepMap µ-Precolumn (Thermo Fisher Scientific) and a 75µm i.d. x50cm 2.1µm particle Acclaim PepMap RSLC analytical column. Loading solvent was 0.1% TFA, analytical solvent A: 0.1% FA and B: ACN+0.1% FA. All separations are carried out at 55 degrees. Samples were loaded at 10µL/min for 5 mins in loading solvent before beginning the analytical gradient. For High pH RP fractions a gradient of 3-5.6% B over 4 mins, 5.6 – 32%B over 162mins, followed by a 5 minute wash at 80%B and a 5 minute wash at 90%B and equilibration at 3%B for 5mins. During the gradient the Orbitrap Fusion mass spectrometer (Thermo Fisher Scientific) was set to acquire spectra.

Data Processing

All Raw files were searched by Mascot within Proteome Discoverer 2.1 (Thermo Fisher Scientific) against the Swissprot Human database and a database of common contaminants.

For TMT labelled samples the search parameters were as follows. Enzyme: Trypsin. MS1 tol: 10ppm. MS2 tol: 0.6Da. Fixed modifications: Carbamidomethyl Cysteine, TMT peptide N-termini and Lysine. Variable modification oxidised methionine. MS3 reporter ion tol: 20ppm, most confident centroid. Mascot Percolator was used to calculate PSM FDR.

Search results were further processed and filtered as follows: Peptides below a percolator FDR of 0.01% and proteins below the 0.01% protein FDR (calculated from a built in decoy database search) were rejected. Protein groups were then generated using the strict parsimony principle. Peptides both unique and razor with a co-isolation threshold of 50 and an average s/n threshold of 10 were used for quantification and a normalisation of these values to the total peptide amount in each channel was applied. Instances where a protein was identified but not quantified in all channels were rejected from further analysis. "Scaled" abundances of proteins provided by Proteome Discoverer were used to derive ratios of abundance.

Bentz, G.L., Jarquin-Pardo, M., Chan, G., Smith, M.S., Sinzger, C., and Yurochko, A.D. (2006). Human cytomegalovirus (HCMV) infection of endothelial cells promotes naive monocyte extravasation and transfer of productive virus to enhance hematogenous dissemination of HCMV. J Virol *80*, 11539-11555.
Butler, L.M., Jeffery, H.C., Wheat, R.L., Rae, P.C., Townsend, K., Alkharsah, K.R., Schulz, T.F., Nash, G.B., and Blackbourn, D.J. (2011). Kaposi's sarcoma-associated herpesvirus infection of endothelial cells inhibits neutrophil recruitment through an interleukin-6-dependent mechanism: a new paradigm for viral immune evasion. J Virol *85*, 7321-7332.
Chan, G., Nogalski, M.T., Stevenson, E.V., and Yurochko, A.D. (2012). Human cytomegalovirus induction of a unique signalsome during viral entry into monocytes mediates distinct functional changes: a strategy for viral dissemination. J Leukoc Biol *92*, 743-752.
Domnina, L.V., Gelfand, V.I., Ivanova, O.Y., Leonova, E.V., Pletjushkina, O.Y., Vasiliev, J.M., and Gelfand, I.M. (1982). Effects of small doses of cytochalasins on fibroblasts: preferential changes of active edges and focal contacts. Proc Natl Acad Sci U S A *79*, 7754-7757.
Elder, E., Krishna, B., Williamson, J., Aslam, Y., Farahi, N., Wood, A., Romashova, V., Roche, K., Murphy, E, Chilvers, E., Lehner, P., Sinclair, J., Poole, E. (2019). Monocytes latently infected with human cytomegalovirus evade neutrophil killing. iScience.
Krishna, B.A., Lau, B., Jackson, S.E., Wills, M.R., Sinclair, J.H., and Poole, E. (2016). Transient activation of human cytomegalovirus lytic gene expression during latency allows cytotoxic T cell killing of latently infected cells. Sci Rep *6*, 24674.
Krishna, B.A., Poole, E.L., Jackson, S.E., Smit, M.J., Wills, M.R., and Sinclair, J.H. (2017a). Latency-Associated Expression of Human Cytomegalovirus US28 Attenuates Cell Signaling Pathways To Maintain Latent Infection. MBio *8*.
Krishna, B.A., Spiess, K., Poole, E.L., Lau, B., Voigt, S., Kledal, T.N., Rosenkilde, M.M., and Sinclair, J.H. (2017b). Targeting the latent cytomegalovirus reservoir with an antiviral fusion toxin protein. Nat Commun *8*, 14321.
Lau, B., Poole, E., Krishna, B., Sellart, I., Wills, M.R., Murphy, E., and Sinclair, J. (2016). The Expression of Human Cytomegalovirus MicroRNA MiR-UL148D during Latent Infection in Primary Myeloid Cells Inhibits Activin A-triggered Secretion of IL-6. Sci Rep *6*, 31205.
Mason, G.M., Poole, E., Sissons, J.G., Wills, M.R., and Sinclair, J.H. (2012). Human cytomegalovirus latency alters the cellular secretome, inducing cluster of differentiation (CD)4+ T-cell migration and suppression of effector function. Proc Natl Acad Sci U S A.
Melak, M., Plessner, M., and Grosse, R. (2017). Actin visualization at a glance. J Cell Sci *130*, 525-530.
Poole, E., Avdic, S., Hodkinson, J., Jackson, S., Wills, M., Slobedman, B., and Sinclair, J. (2014a). Latency-associated viral interleukin-10 (IL-10) encoded by human cytomegalovirus modulates cellular IL-10 and CCL8 Secretion during latent infection through changes in the cellular microRNA hsa-miR-92a. J Virol *88*, 13947-13955.
Poole, E., Bain, M., Teague, L., Takei, Y., Laskey, R., and Sinclair, J. (2012). The cellular protein MCM3AP is required for inhibition of cellular DNA synthesis by the IE86 protein of human cytomegalovirus. PLoS One *7*, e45686.
Poole, E., Juss, J.K., Krishna, B., Herre, J., Chilvers, E.R., and Sinclair, J. (2014b). Alveolar macrophages isolated directly from HCMV seropositive individuals are sites of HCMV reactivation in vivo. J Infect Dis.
Poole, E., King, C.A., Sinclair, J.H., and Alcami, A. (2006). The UL144 gene product of human cytomegalovirus activates NFkappaB via a TRAF6-dependent mechanism. Embo J *25*, 4390-4399.
Zhao, J., Mitrofan, C.G., Appleby, S.L., Morrell, N.W., and Lever, A.M. (2016). Disrupted Endothelial Cell Layer and Exposed Extracellular Matrix Proteins Promote Capture of Late Outgrowth Endothelial Progenitor Cells. Stem Cells Int *2016*, 1406304.
